# Equivalence and non-inferiority trials in the evaluation of non-pharmacological interventions: rationale, challenges and recommendations

**DOI:** 10.1136/bmjopen-2025-102996

**Published:** 2025-08-31

**Authors:** Erland Axelsson

**Affiliations:** 1 Division of Family Medicine and Primary Care, Department of Neurobiology, Care Sciences and Society, Karolinska Institutet, Stockholm, Sweden; 2 Liljeholmen University Primary Health Care Centre, Academic Primary Health Care Centre, Region Stockholm, Stockholm, Sweden

**Keywords:** Clinical trials, STATISTICS & RESEARCH METHODS, EPIDEMIOLOGY

## Abstract

Direct comparisons of non-pharmacological interventions are becoming increasingly common. Many randomised clinical trials are designed to assess whether the effect of one intervention is either equivalent to, or not inferior to, that of a criterion standard. This article provides an accessible introduction to such equivalence and non-inferiority designs. Topics covered include the choice of hypothesis and comparator, the choice of equivalence or non-inferiority margin and the benefits and drawbacks of common methods of analysis. While equivalence and non-inferiority trials offer unique possibilities, there are also challenges. Some of these are particularly pronounced in the non-pharmacological setting, such as the difficulty in establishing margins informed by placebo effects and structural barriers to achieving sufficient statistical power. Under-recognised general threats to the equivalence and non-inferiority designs are also discussed, including the threat of poor intervention delivery leading to washed-out effects, the especially pronounced threat of rhetorical ‘spin’ in the reporting of inconclusive findings and the threat of increasingly indirect evidence due to the sequential change of criterion standards. Implications for the non-pharmacological field are brought to the reader’s attention, and suggestions are given to improve methodological rigour.

**Summary points**

- Equivalence and non-inferiority trials are becoming increasingly common in the evaluation of non-pharmacological interventions.
- This article summarises key methodological choices and threats to such study designs.
- Empirically informed clinical reasoning is needed to establish sound equivalence and non-inferiority margins, and a priori statistical power analyses should mirror ensuing analyses.
- Misleading conclusions can also result from poorly delivered interventions, rhetorical ‘spin’ in the interpretation of inconclusive findings or indirect evidence due to sequential change of criterion standards.

Non-pharmacological research increasingly often involves direct comparisons of active interventions in randomised clinical trials. Traditionally, the vast majority of such direct comparisons have taken place in so-called superiority trials, where the idea is to determine whether one intervention (A) is more effective than another (B). Sometimes though, rather than to determine whether A is more effective than B, it could be more useful to determine whether the effects of A are *sufficiently similar* to those of B. Commonly, the idea is to compare a new and promising intervention (A), perhaps one that demands less resources or results in less negative effects, to a criterion standard (B). Should the effects of the novel intervention be sufficiently similar to those of the criterion standard, potentially, the novel intervention could be a better choice. For example, the effect of a novel non-invasive procedure may be compared with that of an established surgical intervention,^1^ the effect of a more easily disseminated online psychological treatment may be compared with traditional face-to-face psychotherapy^2^ and the effect of intense interval exercise may be compared with that of more time-consuming continuous exercise.^3^

There are two variations on this type of clinical trial: First, there is the *equivalence trial* which is set up to determine if the effect of one intervention is, within a certain margin, similar to that of another. That is: *Is the effect of intervention A equivalent to that of intervention B?* Second, there is the *non-inferiority trial* which is set up to evaluate if one intervention performs at least nearly as well as another, within a specified margin. That is: *Is the effect of intervention A non-inferior to that of intervention B?* Equivalence and non-inferiority trials have much in common. While many methodological concerns relevant for equivalence and non-inferiority trials are equally important for superiority trials, certain threats to trial design are also more pronounced. This article focuses on equivalence and non-inferiority trials, and design aspects that are of particular importance for these, as employed in the evaluation of non-pharmacological interventions.

## Fundamental rationale

Most of medical research relies on null hypothesis significance testing.^4^ This type of statistical analysis focuses on the probability of obtaining an observed outcome, or a more extreme outcome, should the null hypothesis (H_0_) be true. As alluded to above, most clinical trials are not equivalence or non-inferiority trials, but aim to test for an expected difference in effect. In such a clinical trial where participants are assigned to one of two interventions, the typical null hypothesis (H_0_) would be that the two interventions are (generally) equally effective. Assuming, hypothetically, that the two interventions are equally effective (H_0_), if a result is found to be highly improbable (usually p<0.05 or p<0.025), this is said to be statistically significant. The hypothesis of there being no difference (H_0_) can thus be rejected, which is in indirect support of the alternative hypothesis (H_1_) that there is a (general, true) difference between the two interventions. This is commonly referred to as a *superiority trial*.

Equivalence and non-inferiority trials do not follow the same logic. In an equivalence or non-inferiority trial, the expected difference in effect between two interventions is typically small or zero. For example, the investigator may expect a new and more simple surgical procedure to have the same, or roughly the same, effects as a gold standard procedure. Within the framework of traditional null hypothesis (superiority) testing, this would imply that the null hypothesis is in fact true. Under such a scenario, a conventional significance test is of little use. Even though a traditional test that falls short of significance (usually because p≥0.05) indicates that H_0_ cannot be rejected, crucially, this does not directly corroborate H_0_ because the lack of a significant effect could also be the result of insufficient statistical power. In principle, no matter how large the study, it is always possible to argue that the lack of a significant difference between two interventions is merely due to poor precision (lack of statistical power) as opposed to the lack of a true (expected) effect.^5^

**Figure 1:**
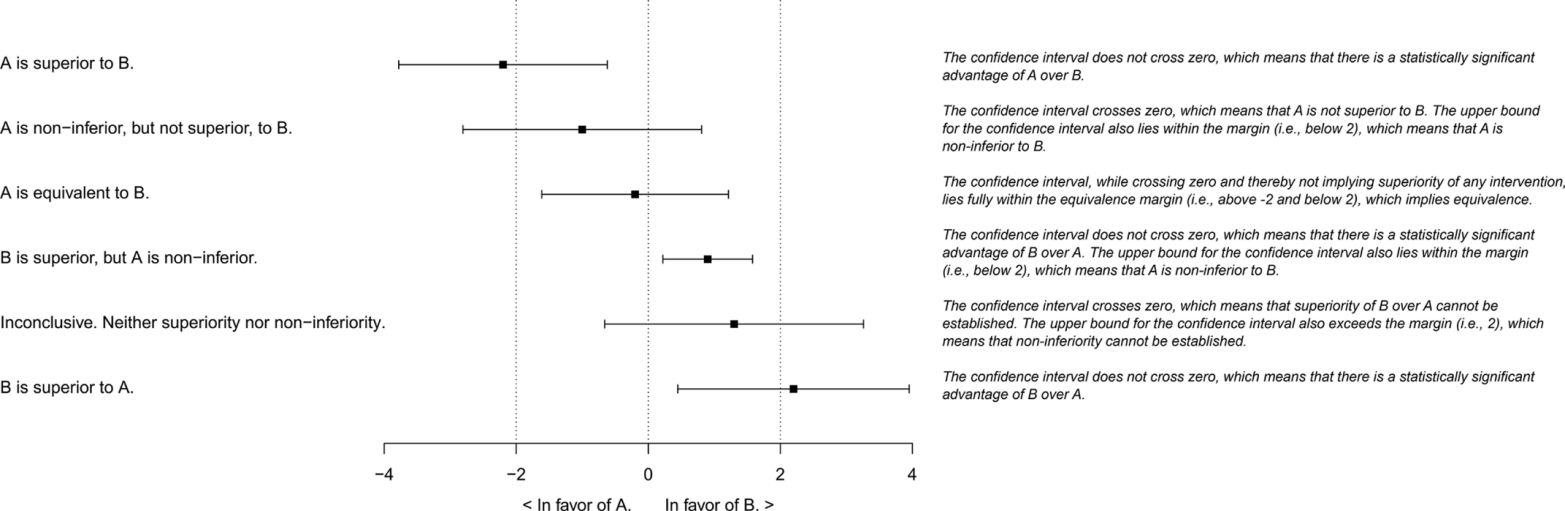
Conventional analytical framework for equivalence and non-inferiority trials, where intervention A is compared with intervention B, as advocated in the 2012 CONSORT extension for the reporting of non-inferiority and equivalence trials.^17^ Note that this framework is based on the presumption that A is the intervention of primary interest, and B the reference standard. The outcome here denoted ’inconclusive’ could also be said to indicate that B is non-inferior to A, which could be important to acknowledge in order to preserve symmetry, for example, in an equivalence trial evaluating two roughly equally established interventions.

Equivalence and non-inferiority trials address this problem by the introduction of an *equivalence-* or *non-inferiority margin*. This is a predefined criterion for *how much* two interventions would have to differ in order for this to be considered somehow relevant. A test for equivalence is one where the researcher determines whether the bounds for the confidence interval (CI) of the difference between the two interventions lie within the equivalence margin. That is to say, while it is still not possible to say with absolute certainty that the difference in effect is zero or negligible, the researcher can say that, with sufficient certainty, the true value does not appear to lie beyond the margin that would make it important—in either direction. Analogously, a test for non-inferiority focuses on whether the upper bound for the CI—the limit that is in favour of the established standard comparator—lies within the non-inferiority margin. Should this be the case, with sufficient certainty, this would mean that there does not appear to be a true advantage of the standard comparator, large enough to be important. A series of possible outcomes (theoretical scenarios) and their typical interpretation is given in figure 1. Some of these have counter-intuitive interpretations. One example is that it is possible for a trial to be indicative of statistical superiority and non-inferiority at the same time. One way of thinking about this could be that statistical and practical significance are distinct,^6^ and that even though there is a true difference in effect, this may still be small enough, with sufficient certainty, to be unimportant.

## Key design considerations

### Premise of a credible criterion standard

As alluded to above, the premise of an equivalence or non-inferiority trial is that the effect of a new intervention is compared with that of a criterion standard. It is important to recognise that this logic presupposes that there *exists* a meaningful criterion standard, such that should equivalence or non-inferiority be established, there is rich evidence in support of the criterion standard that is now also in indirect support for the newer intervention. Not all medical indications are associated with such a criterion standard intervention. Establishing equivalence or non-inferiority in the comparison of two new and experimental therapies, both without a noteworthy evidence base, would typically do little to advance knowledge and would often constitute poor use of resources. A key question for the investigator considering the equivalence or non-inferiority design is therefore whether there exists a meaningful, well-established, criterion standard for the medical indication of interest. Unless such a standard can be identified, it is often more cost-efficient to proceed with superiority trials to further evaluate the effects of the new intervention, which could thereby become criterion standard.

Critics have argued that equivalence and non-inferiority trials increase the risk of a slippery slope development where, based on successive clinical trials, increasingly smaller effects are considered ‘good enough’ (a phenomenon sometimes referred to as ‘biocreep’ or ‘technocreep’).^7^ In other words, should separate trials establish that intervention C is non-inferior to B, and that B is non-inferior to A, it would be tempting to conclude that C is non-inferior to A. This, however, may not be the case because the difference between C and A may in fact exceed the non-inferiority margin. This threat of sequential change of criterion standards highlights the importance of being relatively conservative in the choice of criterion standard. In the reporting of equivalence and non-inferiority trials, the motivation for choice of criterion standard should always be clearly reported.

### Choice of equivalence or non-inferiority margin

The equivalence or non-inferiority margin, usually denoted Δ, represents the largest difference in effect between two interventions that would be acceptable. That is, in the equivalence trial, the equivalence margin stands for the maximum difference between the interventions that would still be considered indicative of equivalence. Analogously, in a non-inferiority trial, the non-inferiority margin stands for the most substantial difference in favour of the criterion standard that would still be indicative of non-inferiority. The choice of an equivalence or non-inferiority margin should be informed both by empirical evidence and clinical judgement. In certain fields, clinical trials may address identical or similar research questions, with similar outcomes and stakeholders in mind, which could allow for a consensus on what effect size constitutes a suitable equivalence or non-inferiority margin. Typically, however, there is no such clear consensus. In order to decide on a margin, the investigator may ask questions such as *What would be the smallest difference between these interventions that would warrant disregarding the novel intervention in favour of the criterion standard?*, and *In light of the advantages of the new intervention, how large an advantage would be acceptable for the criterion standard?*

Sometimes, the equivalence or non-inferiority margin can be informed by previous attempts at estimating the smallest difference of clinical importance for a particular outcome (such as the smallest clinically meaningful difference in muscle strength, or the smallest clinically meaningful difference in points on a scale measuring health-related quality of life). Such a smallest conceivable difference that would be of clinical importance on a particular outcome is commonly referred to as the *minimal clinically important difference (MCID*). The MCID for an outcome can be estimated in many ways, such as on the basis of input from patients, input from clinicians, expert consensus or assumptions about the standardised effect size.^8^ For example, patients or clinicians may be asked to directly report what they would consider the smallest meaningful improvement on a particular outcome. Another method of establishing the MCID could be to relate one outcome measure (such as a novel test of physical strength) to a more established measure (such as another, more established, indicator of physical strength) for which the MCID has already been established. According to a 2008 review, the MCID for patient-reported outcomes (such as standardised questionnaires) typically approximates a standardised mean difference of 0.25–0.50.^9^ This being said, it is important to note that whereas the equivalence or non-inferiority margin always refers to a difference between two interventions on an aggregate group level, the MCID is sometimes rather intended as an indicator on the level of the individual. This implies that the MCID may not always be relevant for decisions on an aggregate basis. In many cases, what constitutes a trivial and unimportant effect—for example, a small improvement in a performance test—on the level of the individual may well be important in terms of group averages (and vice versa). Importantly, decisions about equivalence and non-inferiority on the aggregate level may also take into consideration such concerns as the expected distribution of effects, other benefits of the novel treatment, the resources available and so on.

Another line of reasoning that often informs the choice of equivalence or non-inferiority margin is that most equivalence and non-inferiority trials do not include a rudimentary control group, such as a waitlist or pill placebo condition. It is therefore not possible to conclude from the trial alone that any of the interventions evaluated have effects beyond those of mere placebo responses, the natural course of a condition or even regression towards the mean.^10^ In order to be reasonably certain that the novel intervention has effects that go beyond such rudimentary processes, a common strategy is to ensure that the equivalence or non-inferiority margin lies sufficiently far from the effects expected from a standard rudimentary control group. A widespread guideline for this has been to require margins not to lie beyond half (0.5 times) the lower bound of the 95% CI for the pooled difference between the criterion standard treatment and a rudimentary control group, as based on meta-analysis.^11^ In pharmacological research, most commonly, a meta-analysis vs pill placebo is used to ensure this. Should, for example, a meta-analysis of placebo-controlled effects of the criterion standard intervention present a CI lower bound of 5 on the outcome of interest, the investigator would be expected to ensure that a non-inferiority margin does not allow for novel interventions to be classified as non-inferior if their effect lies more than 2.5 units below the criterion standard.

In much of non-pharmacological research, this type of placebo control is not as straightforward. This is for two reasons. First, in certain strains of non-pharmacological research, it is less clear which aspects of the intervention are to be considered ‘active’ or analogous to the active molecule or substance in pharmacotherapy. For example, it may not be entirely clear which aspects of a certain type of psychotherapy are to be considered specific to that therapy.^12^ Second, there is often a practical problem in that designing placebo controls can be more difficult. In pharmacological research, pill placebo controls can often be implemented with relative ease so as to capture the effects of attention from a caregiver, engagement in a clearly structured intervention and expectations effects as well as conditioned placebo and nocebo responses. In order to capture the corresponding aspects of non-pharmacological interventions, more innovation may be required, and achieving blinding can be more difficult. For example, an investigator interested in the placebo component of acupuncture may be required to develop a form of sham acupuncture complete with non-penetrating needles and a standardised procedure to ensure that these are placed exclusively on non-acupuncture spots.^13^ In certain cases, it can also be ethically problematic to construe a placebo procedure, such as in the field of surgery.^14^ In research fields where there is a lack of widespread and convincing placebo controls, it is nevertheless often possible to ensure that the equivalence or non-inferiority margin lies sufficiently far from the expected effect of very rudimentary procedures, such as waitlists or minimal interventions, to ensure that should the intervention under study be deemed ‘equivalent’ or ‘non-inferior’, the effect of the intervention should also not be trivial.

A third strategy to inform the choice of an equivalence or non-inferiority margin could be to consult previous superiority trials, where judgments have been passed concerning the clinical significance of between-group effects. This method is based on the presumption that the equivalence or non-inferiority margin should mirror the criterion for clinical significance—that is, whether findings are considered important or not—that is employed in superiority trials of the same research field. For example, if researchers typically regard a superior effect with a magnitude of *d*=0.15 to be noteworthy when observed in a superiority trial, it would be irrational to regard group differences of a similar magnitude to be unimportant in equivalence or non-inferiority trials.

In summary, the equivalence or non-inferiority margin should be chosen based on careful consideration. Aspects commonly informing this choice include the MCID, typical effects of rudimentary control conditions and the wider view of which effects are to be considered clinically relevant in the research field as a whole. Ultimately, however, there exists no easy rule-of-thumb criterion that can be applied to ensure that equivalence and non-inferiority margins are appropriate across all clinical trials and situations. The investigator needs to take the particular situation into consideration, including possible effects of trial outcomes for patients, decision makers and other relevant entities. In study protocols and journal articles, the researcher’s rationale for the choice of equivalence or non-inferiority margin should always be clearly reported.

### Intention-to-treat versus per-protocol analysis

Randomised controlled trials (RCTs) are widely regarded as the strongest study design for evaluating medical procedures. RCTs are based on the allocation of participants to conditions—that is, one medical procedure rather than another—by means of randomisation. Because the interventions (conditions) have been allocated entirely by chance, there is no confounding of the effect of intervention allocation (A or B) on the outcome. In other words, if two conditions (A and B) deviate in outcome, the point of the randomised controlled design is that there is no alternative explanation—excluding baseline differences produced by mere chance and the systematic loss of data—other than a causal effect of condition (intervention allocation) on the outcome.

In order to enable such a strong line of reasoning, it is necessary to analyse the outcome of an RCT according to the *intention-to-treat* principle. This means that all participants are analysed in accordance with the condition (intervention) to which they were allocated in the trial, regardless of their subsequent adherence to the protocol, including dropout status and their rate of missing data. Most randomised controlled superiority trials are recommended to report such an intention-to-treat analysis as their primary outcome because excluding participants from the analysis could lead to selection or sampling effects and reintroduces the threat of confounding.^15^

For equivalence and non-inferiority trials, this is still true. However, and importantly, should the integrity of the protocol be compromised—for example, in terms of poor adherence to the protocol, inappropriate last-observation-carried-forward imputation of missing values, or an unreasonably short period of observation—this would inevitably bias the intention-to-treat analysis toward non-inferiority or equivalence. That is to say, true effect differences run the risk of being ‘washed out’ if interventions are delivered in a suboptimal manner. Unlike a superiority trial, in an equivalence or non-inferiority trial, the investigator typically expects—and sometimes outright hypothesises—that the effect will be similar in all conditions of the experiment. This implies that, unlike in the typical superiority trial, the investigator is likely to have something to gain from not fully implementing the intended protocol. This highlights the importance of having guardrails in place to ensure that an adequate level of competence, structural conditions and adherence to key procedures is maintained. For a further discussion of such guardrails, see *Difficulty in maintaining adequate integrity of the protocol* below.

Due to the susceptibility of equivalence or non-inferiority trials to show the expected outcome merely due to poor competence, structural conditions or questionable adherence, one line of argument is that equivalence and non-inferiority trials should combine intention-to-treat with *per-protocol* analysis; the analysis of treatment completers only.^11^ Unlike the intention-to-treat analysis, per-protocol analysis is not susceptible to deviations from the study protocol because this type of analysis is based exclusively on data from participants who completed the intervention that they were assigned to, or followed all key procedures, in the intended manner. The point of combining intention-to-treat with per-protocol could thus be that while the former enables a strict evaluation of the causal effect of condition (intervention allocation) on the outcome, the latter enables an evaluation of treatment effects for those who actually received the intended intervention. Unlike an intention-to-treat analysis, however, the per-protocol analysis is susceptible to confounding. Participants who adhered fully to all procedures, or completed a particular intervention, may, for example, be found to have been particularly highly educated, or had a lower symptom burden than the average participant.

Another line of argument, that goes against the practice of per-protocol analysis, would be that rather than to conduct such a separate analysis focusing on treatment completers only, equivalence and non-inferiority trials should strive towards ensuring a level of intervention integrity that is relevant for the clinical decision at hand (for example, the level of adherence that is typical in clinical care) while maintaining sufficient power of intention-to-treat analyses also given a reasonable level of non-adherence.^16^ Though establishing this reasonable level of adherence and achieving sufficient statistical power could provide a challenge, an advantage of this approach could be that there is little threat of confounding.

Ultimately, the validity of the statistical analysis can only be assessed in relation to the aims of the specific clinical trial, and the precise research question that is to be addressed—including the precise population of interest. In certain situations, it may not be practically or ethically possible to conduct a randomised controlled trial where experimental control is fully maintained. But to the degree that experimental control can be maintained while addressing the research question of interest, this is always preferable. The choice of primary analysis, and the basis for this decision, should be preregistered in the study protocol.

### Coverage of confidence intervals

In mainstream frequentist statistics, α refers to the type I error rate, ie, the probability of incorrectly rejecting the null hypothesis (H_0_). Medical researchers usually employ an α of 5%. This means that, assuming standard assumptions such as random sampling from the population of interest, should a clinical trial be repeated indefinitely, the true effect will lie within the CI in 95% of these replications. A smaller α implies a lower willingness to accept type I errors, and therefore a wider CI which is more likely to include the true effect in each replication.

As described above, in equivalence and non-inferiority trials, the idea is to relate the difference between two interventions—and the CI for this difference—to the equivalence or non-inferiority margin (figure 1). One important design aspect is therefore to decide on the characteristics of this CI. Just as in a superiority trial, a smaller α, and a wider CI, implies a more rigorous analysis. Suppose, for example, that two separate equivalence trials evaluate the same two physiotherapies, with the same equivalence margin, the same sample size and observe the same treatment effect. If one of these trials employs a smaller α, its CI will be wider. Therefore, this first trial may indicate that the outcome is inconclusive, while the other trial may be indicative of equivalence, solely due to the adaption of different αs.

In a non-inferiority trial, an added layer of complexity is also whether a one- or two-sided test is to be preferred. Just as in a conventional superiority trial, a one-sided test is less stringent than a two-sided test if both adhere to the same confidence level and focus on the same tail of the distribution. The widespread CONSORT guidelines^17^ suggest that a two-sided CI would be appropriate for most non-inferiority trials, but that it can also be reasonable to employ a one-sided CI in certain situations. Such a one-sided test could be based either on an α of 2.5% (corresponding to the upper bound of a 95% two-sided interval) or an α of 5% (corresponding to the upper bound of a 90% two-sided interval). Some entities such as the European Medicines Agency (EMA)^18^ have argued for 95% two-sided intervals being the only acceptable choice in equivalence and non-inferiority trials, primarily in order to maintain consistency over trial designs, and also because should the analysis be indicative of inferiority (in favour of the criterion standard), a conventional significance test of superiority of the comparator can be based on the same CI. The type of CI—α, and whether the test is one- or two-sided—used for evaluating equivalence or non-inferiority should be preregistered in the study protocol.

### Appropriate a priori power analysis

A statistical power analysis is an important step of designing any clinical trial. For the equivalence or non-inferiority trial, the main focus of such an analysis is to ensure that should the effect of two interventions be equivalent, or one intervention be non-inferior to another, with reasonable probability, the trial will demonstrate this. Importantly, a conventional power analysis focusing on a test of superiority (trying to determine if A is superior to B) is of little relevance in determining whether a clinical trial is of sufficient size to enable a meaningful analysis of equivalence (if A is equivalent to B) or non-inferiority (if A is not inferior to B). The power analysis must be informed by the expected effects, the employed equivalence or non-inferiority margin and the precise intended method of analysis including the type of CI.

The level of power that is desirable is a matter of debate. Whereas for superiority trials, it is common to design for 80% power, a common though not universal recommendation for equivalence and non-inferiority trials is to aim for a slightly higher target of 90%, motivated by the fact that there are usually high stakes involved when comparing newer therapies to an established criterion standard.^19^ One often overlooked aspect of estimating the power of equivalence and non-inferiority trials is that even though a difference in the effect of two interventions could be expected to fall within the equivalence or non-inferiority margin, ie, not be of any practical importance, the same difference could also, simultaneously, be expected to deviate from zero. This has implications for statistical power. The closer the true (expected) difference in effect lies to the equivalence or non-inferiority margin, the more study participants are needed to distinguish between the true effect and the margin.

Another important aspect of longitudinal data analyses is the appropriate management of missing data. This is a complex matter that will not be addressed at any depth here.^20^ Worthy of particular note, however, is that the widely discouraged yet relatively common method of imputing missing values with the last observed value can be especially detrimental for equivalence and non-inferiority trials.^21^ Not only does the imputation of single values lead to artificially reduced variance and thereby narrow confidence intervals, but the point estimate will often be biased towards the expected outcome of a zero or negligible difference in the effect of two interventions. In summary, it is crucial that sufficient statistical power is ensured through an a priori power analysis which takes the expected effects and precise method of analysis into consideration. This analysis and the target sample size should be preregistered.

### Reporting outcomes without ‘spin’

In equivalence and non-inferiority trials, should an insufficient number of participants be included, or the variance in the outcome be higher than expected, it may not be possible to establish superiority, nor equivalence or non-inferiority. As can be seen in figure 1, the most widely recommended approach to this situation is to conclude that the trial was *inconclusive*. Surveys of the existing literature have concluded, however, that it is relatively common for the outcome of at least non-inferiority trials to be reported in a biased manner. Perhaps the most widely debated example of this is that an inconclusive result—reflective of a situation where in fact it is not possible to, given the desired precision, deduce anything about the relative effects of two interventions—may be illegitimately reframed as somehow supportive of non-inferiority, or even equivalence. This and similar practices, though certainly not unique to equivalence and non-inferiority trials, are commonly referred to as ‘spin’. This appears to be especially common when the intervention is described as new and without non-profit funding.^22^ The problem is widespread, however.^23^ Preregistration offers an opportunity to decide a priori on an appropriate course of action conditioned on different potential outcomes.

## Challenges especially pronounced in the non-pharmacological setting

### Mismatch between costs and resources

Clinical trials can be costly, including in non-pharmacological research. In order to study the smallest effects of practical importance, it may be necessary to recruit hundreds or even thousands of participants. Non-pharmacological trials may require such a large sample to undergo a complex protocol including, for example, surgical procedures, daily training with a physiotherapist, months of sessions with a psychotherapist or extensive lifestyle changes. At the same time, the academic system is set up in a manner that rewards rapid completion of studies and the production of scientific manuscripts. Non-pharmacological research is not always a priority, and when there is a shortage of funding, it is understandably tempting for investigators to conduct underpowered trials either in the hope of improving knowledge or to pursue career goals.^24^ But underpowered trials are of little use in informing clinical practice. Insufficiently powered or conducted trials may mislead, not only because results are different from those that had been obtained from best practice clinical trials (for example, resulting in inconclusive results rather than equivalence or non-inferiority), but also because poor trials reduce the incentive for the completion of other, more trustworthy, designs.^25^ As in the case of superiority trials, meta-analysis can be used to pool estimates from several equivalence or non-inferiority trials and to benchmark these against thresholds for practical significance. That said, even meta-analyses can be biased if not all original studies are openly reported.^26^

### Challenges in achieving blinding

In non-pharmacological trials, it is often unrealistic to achieve full blinding in the sense that patients are unaware of the processes of an intervention, such as the type of exercises in a physiotherapy. It is usually the case, however, that non-pharmacological trials can be blinded to a certain degree.^27^ With regard to intervention allocation and content, study personnel and patients should ideally be unblinded only on a need-to-know basis, when this is justified by the research question. For example, while a physiotherapist usually has to know what kind of therapy is supposed to be delivered for a specific patient, another clinician could evaluate the patients’ symptoms after therapy without knowledge of which treatment was given. Blinding can also play a role in the data analysis phase, where the individual performing the statistical analyses can be blinded to the interventions in order not to be influenced in choice of procedures or key interpretations. Even when patients need to be aware of certain aspects of the intervention, the influence of bias could be reduced by patients remaining blind to other aspects of the trial protocol, such as the other intervention that is evaluated, the hypotheses of the trial, key findings of previous trials (when motivated and not ethically problematic) and so on. In order to better understand the role of patient expectancies, this can also be measured and reported. Another possibility is to measure the degree to which blinding was achieved and assess in secondary analyses how much this appeared to influence the results of the trial.

### Difficulty in maintaining adequate integrity of the protocol

It is common to make a distinction between the *internal validity* of a clinical trial—if the design allows for conclusions about causality—and the *external validity*—if findings can be generalised to real-world situations. For example, should an equivalence trial comparing two exercise programmes make use of a highly reliable repeated measurement method, this would speak for internal validity, meaning a rigid evaluation of effects. But if this method necessitates recurrent and extensive support from a clinician, the external validity of the trial could be poor, meaning that effects may not generalise to the routine care environment where the level of support is more limited. Oftentimes, there is this type of trade-off between internal and external validity. In non-pharmacological equivalence and non-inferiority trials, the internal vs external validity trade-off is brought to the centre of attention. On the one hand, ensuring the integrity of the trial protocol is important in order not to ‘wash out’ the effect of interventions, thus biasing the between-group effect toward zero. This can be a challenge, especially if the intervention is lengthy or complex, which is often the case. On the other hand, an undue strict study protocol can lead to questionable external validity. Establishing that an intervention was delivered in an adequate manner usually boils down to demonstrating an appropriate level of (i) *competence*, (ii) *structural conditions* and (iii) *adherence to key procedures*. Competence implies making sure that clinicians or other personnel working in the trial have received sufficient training and possess the relevant personal characteristics that enable them to deliver the intervention. This can be ensured through careful recruitment, education and recurrent supervision. Structural conditions imply all sorts of infrastructure, such as, for example, facilities and materials necessary to conduct surgical procedures, or the necessary equipment for patients to engage in challenging physical exercise. This also includes the format such as the length of the intervention, frequency of communication, mode of manipulation and so on. Adequate structural conditions can be ensured through careful planning and the acquisition of necessary resources. Third, adherence to key procedures can be promoted, for example, through standardised procedures, and by means of checklists and quality checks. For example, surgical procedures or sessions in psychotherapy may be recorded and reviewed by an external party, in accordance with predefined criteria. This being said, procedures to ensure adequate adherence are required not to imply unacceptable threats to external validity. Just as in any superiority trial, should the clinicians be experts of a kind rarely seen in the routine clinical setting, the structural resources lie far beyond what would be attainable in the clinic, or adherence to the study protocol be extreme to the point where the trial says very little about clinicians’ and patients’ behaviour in the real world, this could jeopardise generalisability. To be informative for routine care, the evaluated interventions should be delivered in the manner that these would be delivered there, and in order for internal validity to be maintained, trials need to be powered based on this presumption.

## Summary and concluding remarks

In summary, certain design considerations, including the premise of a credible criterion standard and the choice of an appropriate equivalence or non-inferiority margin, are relevant for all equivalence and non-inferiority trials. Certain design considerations are also especially relevant for non-pharmacological equivalence and non-inferiority trials. Such aspects include the challenge of maintaining and demonstrating blinding and adequate integrity of the protocol, often with limited resources. A more exhaustive summary of key fundamental design concerns, common potential malpractices and best practice recommendations is provided in table 1.

**Table 1:** Fundamental design aspects of non-pharmacological equivalence and non-inferiority trials

Design aspect	Common potential malpractice	Best practice recommendation
Credible criterion standard	Use of a comparator with limited empirical support, either in general or for the indication of main interest	An established criterion standard for the indication is a requirement for an equivalence or non-inferiority trial
Equivalence or non-inferiority margin	Margin either lacks a clear justification, lies close to rudimentary effects or is indicative of equivalence or non-inferiority even in the presence of effects that would be regarded as important in a superiority trial Margin is not preregistered	Margin presented with clear justification, lies sufficiently far from rudimentary effects and is not indicative of equivalence or non-inferiority in the presence of effects that would be regarded as important in a superiority trial Margin is preregistered in a public database
Analytical framework	No intention-to-treat analysis even though relevant Unclear specification of CI Problematic management of missing data, for example, complete case analysis or single imputation of missing values with the last observed value Method of analysis is not preregistered, or there is ambiguity about which test that is primary	Intention-to-treat analysis is conducted if relevant, and there is either demonstratable integrity of the protocol, or the addition of sensitivity, such as per-protocol, analyses Explicit specification of CI Appropriate management of missing data Method of analysis is preregistered in a public database, with clear specification of primary test
Statistical power	No a priori power analysis, power analysis is not concerned with the primary test of equivalence or non-inferiority, or power analysis does not account for expected effects, adherence and missing data Power analysis and target sample size are not preregistered or registered in an ambiguous manner	A priori power analysis focuses on the model used for the primary test of equivalence or non-inferiority and accounts for expected effects, adherence and patterns of missing data Power analysis and target sample size are preregistered in a public database, with key modelling assumptions
Integrity of protocol	No concern for integrity of the protocol in relation to interpretation of equivalence or non-inferiority results Competence, structural conditions and adherence are either too beneficial, jeopardising external validity, or too poor, leading to diminished between-group effects	Integrity of the protocol is taken into account in the interpretation of equivalence or non-inferiority results Competence, structural conditions and adherence are demonstrated to be adequate in relation to the research question and settings to which results are generalised
Unbiased reporting	Inconclusive findings are interpreted as indicative of equivalence or non-inferiority, or over-emphasis on secondary, for example, within-group, outcomes	Inconclusive results are clearly acknowledged, without undue over-emphasis on secondary, for example, within-group, outcomes
