# Supplementary material for: Equivalence and non-inferiority trials in the evaluation of non-pharmacological interventions: rationale, challenges and recommendations
Source: BMJ Open. 2025 Aug 31;15(8):e102996. doi: 10.1136/bmjopen-2025-102996 (PMC12406809; doi:10.1136/bmjopen-2025-102996)
Supplement: Supplementary file 1 [file bmjopen-2025-102996.reviewer_comments.pdf]

PEER REVIEW HISTORY

BMJ Open publishes all reviews undertaken for accepted manuscripts. Reviewers are asked to complete a checklist review form and are provided with free text boxes to elaborate on their assessment. These free text comments are reproduced below.

ARTICLE DETAILS

Title (Provisional)

Equivalence and non-inferiority trials in the evaluation of non-pharmacological interventions: rationale, challenges, and recommendations

Authors

Axelsson, Erland

VERSION 1 - REVIEW

**Reviewer**	**1**
**Name**	**Quartagno, Matteo**
**Affiliation**	**University College London**
**Date**	**14-May-2025**
**COI**	**None**

Dear author,

it was a pleasure reading this article. The review is clearly written and informative, and I agree with most of what was written. I have two main comments though, which are quite important in my view:

1) Not sure I find the case for writing such an article really compelling. Most of the article in a standard summary of what non-inferiority trials are. It is well written and can agree on almost everything, but don't think we need right now such an article, and sounds more like a good summary in a book chapter. The section on specific challenges in non-pharma settings does not seem to address such challenging issues to warrant a paper such as this. There are only two points which are quite quick to address, somewhat trivial and do not seem worthy of a whole paper.

2) In terms of the content per se, the one thing I did not find in line with current literature is the discussion of ITT and PP. In the pharma settings the estimands framework is now well established, and I don't see why that should not apply to the non-pharma settings. I personally believe that has helped clarifying many aspects of the trial, and particularly the fact that there is no "biased analysis" per se but only one that is biased "for a specific estimand". I do not think that all NI trials should target a treatment-policy estimand (aka ITT)

or a hypothetical one (aka PP) nor a combination of the two. Each trial should target the right estimand and use an analysis method that is as good as possible at estimating it.

**Reviewer**	**2**
**Name**	**Totton, Nikki**
**Affiliation**	**The University of Sheffield, ScHARR**
**Date**	**08-Jun-2025**
**COI**	**None**

Thank you for this manuscript, I enjoyed reading it. I think it is a nice overview of equivalence and non-inferiority trials.

One overarching comment I have is the lack of references in some places which could be useful to the reader if they wish to look further into a certain aspect, for example referencing some of the more prominent guidelines for setting the margin would be useful. Additionally, adding references for claims such as “A widespread guideline for this has been to require margins not to lie beyond half (0.5 times) the lower bound of the 95% confidence interval for the pooled difference between the criterion standard treatment and a rudimentary control group, as based on meta-analysis” would be useful.

Other minor comments are for clarity for the reader:

1. Page 4: I am not sure on the clarity of the definition for non-inferiority: “Second, there is the non-inferiority trial which is set up to determine whether, within a certain margin, the effect of one intervention is at least as large as the effect of another.” Initially I read this as the difference would be in favour of the intervention as with a superiority trial. Could this be reworded?

2. Page 5: Add Superiority to the “In a clinical trial” for the example to be clear from the outset.

3. Page 6: To the end of “That is to say, while it is still not possible to say with absolute certainty that the difference in effect is zero or negligible, the researcher can say that, with sufficient certainty, the true value does not appear to lie beyond the margin that would make it important. ” Add that this difference is in either direction for clarity that it’s both bounds of the CI.

4. Page 9: “In certain fields, there exists a consensus on what effect size constitutes a suitable equivalence or non-inferiority margin”. Although this is true, guidance suggests each margin should be considered based on the specific intervention, comparator, outcome and benefits and so it’s not recommended that a standard effect size is blanket used for NI trials which would be useful to be mentioned here.

VERSION 1 - AUTHOR RESPONSE

Reviewer 1 general comment:

>>it was a pleasure reading this article. The review is clearly written and informative, and I agree with most of what was written.>>

Response:

This is encouraging, and especially that the manuscript was easy to follow despite the complex topic. This was a key objective.

Reviewer 1 specific comment #1:

>>1) Not sure I find the case for writing such an article really compelling. Most of the article in a standard summary of what non-inferiority trials are. It is well written and can agree on almost everything, but don't think we need right now such an article, and sounds more like a good summary in a book chapter. The section on specific challenges in non-pharma settings does not seem to address such challenging issues to warrant a paper such as this. There are only two points which are quite quick to address, somewhat trivial and do not seem worthy of a whole paper. >>

Response:

I do not agree that this type of “good summary” should be considered beyond the scope of the journal. My understanding is that BMJ Open has a long track record of promoting open science practices, particularly with regard to clinical trials, and that the manuscript under review would align very well with this tradition in that the article aims to promote methodological clarity and transparency in equivalence and non-inferiority trials. Furthermore, BMJ Open has a broad disciplinary focus, with articles from family medicine, surgery, physiotherapy, clinical psychiatry, and paramedical fields etc. – which suggests a suitable platform for discussing methods relevant for nonpharmacological interventions.

Something that I would like to emphasize here is also the intended audience. My impression is that BMJ Open largely targets clinical researchers from the medical professions, with articles that are “relevant to patients and clinicians” (1). I can understand and empathize with Reviewer 1 finding certain elements of the text trivial, but it is also important to keep the intended audience in mind. As I mentioned briefly in the presubmission cover letter, being a clinical researcher (psychologist) myself, I have often been consulted by colleagues with an emerging interest in equivalence and non-inferiority trial designs, and my impression has been that these colleagues have had little opportunity to get basic questions answered

elsewhere. Existing introductory texts are often technical, and lack a nonpharmacological perspective which means that there is no mention for example of the inherent difficulties of referencing placebo effects when choosing a margin, the importance of monitoring and reporting competence and adherence to the intervention, and so on. In order cover such challenges, which are documented in the literature (2), it is necessary to first establish key facts about these study designs, and to address common misconceptions while maintaining a nonpharmacological perspective with suitable such examples and emphasis. For the revised version (R1), in accordance with the feedback from Reviewer 1, the part of the text that concerns nonpharmacological designs specifically has been a little more fleshed out, though fundamentally I believe that introducing terms is a strength of the text, not a weakness.

Reviewer 1 specific comment #2:

>> 2) In terms of the content per se, the one thing I did not find in line with current literature is the discussion of ITT and PP. In the pharma settings the estimands framework is now well established, and I don't see why that should not apply to the non-pharma settings. I personally believe that has helped clarifying many aspects of the trial, and particularly the fact that there is no "biased analysis" per se but only one that is biased "for a specific estimand". I do not think that all NI trials should target a treatment-policy estimand (aka ITT) or a hypothetical one (aka PP) nor a combination of the two. Each trial should target the right estimand and use an analysis method that is as good as possible at estimating it. >>

Response:

I thank Reviewer 1 for this insightful comment and wholeheartedly agree that no statistical analysis is biased per se: the validity of all methods need be assessed in relation to the aims of the research, i.e., the precise research question that is to be addressed – pertaining to a specific population, with a specific scope, and so on. The revised text now acknowledges that (p. 15): “Ultimately, the validity of the statistical analysis can only be assessed in relation to the aims of the specific clinical trial, and the precise research question that is to be addressed – including the precise population of interest. In certain situations, it may not be practically or ethically possible to conduct a randomized controlled trial where experimental control is fully maintained.” I would also like to soften the argument a little bit. There should be no question that, in science, whenever it is practically and ethically possible to maintain experimental control, we should always strive to maintain that experimental control. And this can only be accomplished if we base the analysis on all units randomized, or a subset of these units that is selected either at random or on a characteristic relevant for the research focus, such as baseline gender, that could not be directly or indirectly affected by the interventions. In certain situations, to address certain research questions, this may not be

possible, of course. But when practically and ethically possible, this is simply the strongest design (granted externalities such as sufficient power). I believe there is a risk that even experienced readers may lose sight of this basic fact if too much terminology is used. Considerations such as this may also explain why the CONSORT 2025 workgroup was not able to reach consensus on whether the estimands framework should be widely promoted or not (3). Many readers of medical journals have no or rudimentary knowledge about statistical analysis (4-6). This highlights the importance of accessible language, which I have prioritized in this manuscript.

Reviewer 2 general comment #1:

>> Thank you for this manuscript, I enjoyed reading it. I think it is a nice overview of equivalence and non-inferiority trials. >>

Response:

This was a heartwarming comment, much appreciated.

Reviewer 2 general comment #2:

>>One overarching comment I have is the lack of references in some places which could be useful to the reader if they wish to look further into a certain aspect, for example referencing some of the more prominent guidelines for setting the margin would be useful. Additionally, adding references for claims such as “A widespread guideline for this has been to require margins not to lie beyond half (0.5 times) the lower bound of the 95% confidence interval for the pooled difference between the criterion standard treatment and a rudimentary control group, as based on meta-analysis” would be useful. >>

Response:

Good point. I was originally hesitant to add more references because in my experience non-empirical articles in general medical journals typically do not have that many. I have reviewed the text for opportunities to add more clarifications and references, including the sentence referenced by Reviewer 2, and the reference list has expanded from 22 to 28 entries. While doing so, I have also reviewed all references to ensure that these are specific and preferably easy to follow.

Reviewer 2 specific comment #1:

>>Page 4: I am not sure on the clarity of the definition for non-inferiority: “Second, there is the non-inferiority trial which is set up to determine whether, within a certain margin, the effect of one intervention is at least as large as the effect of another.” Initially I read this as the difference would be in favour of the intervention as with a superiority trial. Could this be reworded?>>

Response:

Good point. Now reads (p. 4): “Second, there is the non-inferiority trial which is set up to evaluate if one intervention performs at least nearly as well as another, within a specified margin.”

Reviewer 2 specific comment #2:

>>Page 5: Add Superiority to the “In a clinical trial” for the example to be clear from the outset.>>

Response:

This passage is now rewritten to introduce the concept of “superiority” and to clarify that the sentence cited by Reviewer 2 refers to that type of trial (p. 5): “As alluded to above, most clinical trials are not equivalence or non-inferiority trials, but aim to test for an expected difference in effect. In such a clinical trial where participants are assigned to one of two interventions, the typical null hypothesis (H0) would be that the two interventions are (generally) equally effective. Assuming, hypothetically, that the two interventions are equally effective (H0), if a result is found to be highly improbable (usually $p<0.05$ or $p<0.025$), this is said to be statistically significant. The hypothesis of there being no difference (H0) can thus be rejected, which is in indirect support of the alternative hypothesis (H1) that there is a (general, true) difference between the two interventions. This is commonly referred to as a superiority trial.”

Reviewer 2 specific comment #3:

>>Page 6: To the end of “That is to say, while it is still not possible to say with absolute certainty that the difference in effect is zero or negligible, the researcher can say that, with sufficient certainty, the true value does not appear to lie beyond the margin that would make it important. ” Add that this difference is in either direction for clarity that it’s both bounds of the CI.>>

Response:

Now reads (p. 6): “That is to say, while it is still not possible to say with absolute certainty that the difference in effect is zero or negligible, the researcher can say that, with sufficient certainty, the true value does not appear to lie beyond the margin that would make it important – in either direction.”

Reviewer 2 specific comment #4:

>>Page 9: “In certain fields, there exists a consensus on what effect size constitutes a suitable equivalence or non-inferiority margin”. Although this is true, guidance suggests each margin should be considered based on the specific intervention, comparator, outcome and benefits and so it’s not recommended that a standard effect size is blanket used for NI trials which would be useful to be mentioned here.>>

Response:

I wholeheartedly agree with these principles, though to some extent I also believe an argument could be made that a field is defined precisely by an overlap in such factors. This passage has now been nuanced (p. 9): “In certain fields, clinical trials may address identical or similar research questions, with similar outcomes and stakeholders in mind, which could allow for a consensus on what effect size constitutes a suitable equivalence or non-inferiority margin. Typically, however, there is no such clear consensus. In order to decide on a margin, the investigator may ask questions such as […]”

VERSION 2 - REVIEW

**Reviewer**	**2**
**Name**	**Totton, Nikki**
**Affiliation**	**The University of Sheffield, ScHARR**
**Date**	**11-Aug-2025**
**COI**	

Thank you for making changes to the manuscript, I have no further comments or questions.
